# Immune Modulation Effects of *Lactobacillus casei* Variety *rhamnosus* on Enterocytes and Intestinal Stem Cells in a 5-FU-Induced Mucositis Mouse Model

**DOI:** 10.1155/2021/3068393

**Published:** 2021-01-25

**Authors:** Chun-Yan Yeung, Jen-Shiu Chiang Chiau, Mei-Lien Cheng, Wai-Tao Chan, Szu-Wen Chang, Chuen-Bin Jiang, Hung-Chang Lee

**Affiliations:** ^1^Department of Pediatric Gastroenterology, Hepatology and Nutrition, MacKay Children's Hospital, Taipei, Taiwan; ^2^Department of Medical Research, MacKay Memorial Hospital, Taipei, Taiwan; ^3^Department of Medicine, MacKay Medical College, New Taipei City, Taiwan

## Abstract

**Background:**

Intestinal mucositis remains one of the most deleterious side effects in cancer patients undergoing chemotherapy. We hypothesize that the probiotics could preserve gut ecology, ameliorate inflammation, and protect epithelia via immune modulations of enterocytes and intestinal stem cells. Our aim is to characterize these changes and the safety of probiotics via a 5-fluorouracil- (5-FU-) induced intestinal mucositis mouse model.

**Methods:**

5-FU-injected BALB/c mice were either orally administrated with saline or probiotic suspension of *Lactobacillus casei* variety *rhamnosus* (Lcr35). Diarrhea scores, serum proinflammatory cytokines, and T-cell subtypes were assessed. Immunostaining analyses for the proliferation of intestinal stem cells CD44 and Ki67 were processed. Samples of blood and internal organs were investigated for bacterial translocation.

**Results:**

Diarrhea was attenuated after oral Lcr35 administration. Serum proinflammatory cytokines were significantly increased in the 5-FU group and were reversed by Lcr35. A tremendous rise of the CD3^+^/CD8^+^ count and a significant decrease of CD3^+^CD4^+^/CD3^+^CD8^+^ ratios were found in the 5-FU group and were both reversed by Lcr35. 5-FU significantly stimulated the expression of CD44 stem cells, and the expression was restored by Lcr35. 5-FU could increase the number of Ki67 proliferative cells. No bacterial translocation was found in this study.

**Conclusions:**

Our results showed that 5-FU caused intestinal inflammation mainly via Th1 and Th17 responses. 5-FU could stimulate stem cells and proliferation cells in a mouse model. We demonstrate chemotherapy could decrease immune competence. Probiotics were shown to modulate the immune response. This is the first study to analyze the immune modulation effects and safety of *Lactobacillus* strain on enterocytes and intestinal stem cells in a mouse model.

## 1. Background

Mucositis is a common and clinically significant side effect of chemotherapy that can affect any portion of the gastrointestinal tract. The incidence of chemotherapy-induced mucositis has been reported as 50–80% of patients treated with high-dose chemotherapy [1–3]. Intestinal mucositis can cause treatment delays, interruptions of anticancer drugs, and increased complication rates [1]. In 2014, the Multinational Association of Supportive Care in Cancer/International Society of Oral Oncology published an updated clinical practice guideline for mucositis and was considered the keystone of prevention and treatment of mucositis [4]. However, the management of intestinal mucositis remains mostly symptomatic at present [5].

In recent years, probiotics had demonstrated therapeutic effects in clinical diseases such as inflammatory bowel disease and chemotherapy-induced mucositis. Because commensal bacteria play pivotal roles in both the innate and adaptive immune systems of the host, intestinal dysbiosis is considered part of the reasons in the pathophysiology of chemotherapy-induced mucositis [6, 7]. Therefore, the normalization of intestinal homeostasis could be an appropriate strategy to improve the status of patients receiving chemotherapy. In recent years, the use of probiotics to alleviate damage to the intestinal mucosa has been supported by clinical consensus [8]. Besides, we previously discovered that various *Lactobacillus* strains could relieve the intestinal barrier damage induced by *Salmonella* lipopolysaccharide [9]. We also demonstrated that *Lactobacillus* strain and a mixture of *Lactobacillus* and *Bifidobacterium* strains could attenuate inflammation and protect epithelia by maintaining the tight junction integrity and reduce the severity of 5-FU-induced intestinal mucositis in a mouse model [10]. Much progress has been made in recent years in terms of understanding the pathological and signaling alterations occurring in the gut subsequence to chemotherapy treatment [11]. In our previous study, we showed that oral probiotic Lcr35 prevented 5-fluorouracil/oxaliplatin-induced intestinal mucositis in colorectal cancer-bearing mice. The putative mechanism might involve modulation of gut microbiota and proinflammatory responses with suppression of intrinsic apoptosis in intestinal injury [12]. Recently, we also successfully demonstrated that the gut microbiota of mice undergoing chemotherapy exhibited a distinct disruption in the bacterial composition. Probiotics did modulate the abundance and diversity of gut microbiota [13].

In this study, we hypothesize that the probiotics could preserve gut ecology, ameliorate inflammation, and protect epithelia by maintaining the tight junction integrity via immune modulations of enterocytes and intestinal stem cells. Our aim is to characterize these changes and to investigate the immune modulation effects and safety of probiotics via a 5-FU-induced intestinal mucositis mouse model. In addition, various serum proinflammatory cytokines and intestinal histological changes would be assessed. We further investigate T-cell subtypes by flow cytometry and the proliferation of intestinal stem cells CD44 and Ki67 by immunostaining analyses in this study.

## 2. Methods

### 2.1. 5-FU Treatment

5-FU (Fluorouracil-TEVA®, Netherlands) was injected intraperitoneally (IP) at a single dose of 30 mg/kg/day on the first day to cause intestinal mucositis and diarrhea as described in our previous study [10]. IP saline was injected as an alternative in the control group. Body weight changes and diarrhea scores were recorded and assessed daily, and the results were compared. We used Bowen's score system to assess diarrhea severity [14]. Severity was classified into four grades according to stool consistency.

### 2.2. Probiotic Preparation

*Lactobacillus casei* variety *rhamnosus* (Lcr35, Antibiophilus®, France) (1 × 10^7^ CFU) was used in this experiment. The probiotic was diluted in sterile saline and administered by oral gavages as described in our previous research [10]. The mice received 100 *μ*l of saline or suspension containing 1 × 10^7^ CFU of the probiotic daily for 5 days. This probiotic strain was chosen because it was widely used clinically in chronic gastrointestinal disorders in our country and has shown promising results in maintaining tight junction integrity in our previous study [9].

### 2.3. Animal Trial

Male BALB/c mice were used in our experiments. They were obtained from Taiwan's National Laboratory Animal Center under a 12 h light/dark cycle with a temperature of 22 ± 1°C and a humidity of 55 ± 10%. All mice were given *ad libitum* access to autoclaved food (Laboratory Autoclavable Rodent Diet 5010) and water. The mice were at the age of about 6 weeks with weight 24 ± 3 g and were randomly assigned as four groups (*n* = 4-5). The mice were injected with saline or 5-FU IP on the first day. Mice in each control group and experimental group were then orally administrated with saline or probiotic suspension of Lcr35 daily. Body weight was measured daily. On day 5 posttreatment, mice were submitted to euthanasia for blood sampling. Mice were treated by inhaled anesthesia by using 2-5% isoflurane for 3 minutes and cardiac puncture for blood collection.

### 2.4. Ethics Statement

Animal studies were approved by the Institutional Animal Care and Use Committee (IACUC) of MacKay Memorial Hospital (MMH-A-S-105-26). IACUC has been accredited, approved, and authorized by the government office, Agriculture and Food Agency Council of Agriculture, Executive Yuan, Taiwan. All methods were performed in accordance with the relevant guidelines and regulations in this animal study.

### 2.5. Cytokines and Flow Cytometry Analysis

Blood samples were collected and centrifuged after sacrifice. The serum was analyzed by the Bio-Plex Pro™ Mouse Cytokine Multi-Plex Panel Kit (Bio-Rad Laboratories, Inc., USA). Targets of cytokines included IL-1*β*, IL-4, IL-6, IL-17A, IFN-*γ*, MCP-1, and TNF-*α*. The results were expressed as pg/ml.

To evaluate the subtypes of T-cells, the peripheral blood monocyte cells (PBMC) were collected from whole blood by using BD FACS™ Lysing Solution. Blood cells were calculated by the complete blood cell count (HEMAVET®). A total of 3 × 10^5^ cells were washed with PBS and resuspended in 1 ml PBS. The suspension (50 *μ*l) was incubated with anti-CD3-PE, CD4-FITC, CD8-PerCP-Cy5.5 (BD Pharmingen™, CA) for 30 min at 4°C and then washed with cold PBS. After incubation, cells were postfixed and permeated with 300 *μ*l of Cell Fix 1x (BD Cytofix/Cytoperm) and were kept at 4°C in the dark. The cells were resuspended in 1 ml PBS. Then, the cells were stained by IL-4-PE and IL-17A-PE for 30 min at 4°C and then washed with cold PBS. Data were recorded using a BD FACSCalibur and analyzed using the BD CellQuest Pro software (both from Becton Dickinson, NJ, USA). The results were multiplied by the percentage of T-cell subtypes and the quantity of leukocytes.

### 2.6. Histological Analysis for Villus Height, Crypt Depth, and Goblet Cells

Jejunal specimens with a 2 cm ring each were collected after sacrifice and were processed and fixed in 10% buffered neutral formalin. Sections were routinely hematoxylin-eosin stained for tissue morphology. Periodic acid-Schiff and Alcian blue- (PAS+AB-) stained goblet cells were expressed as the number of goblet cells per villus-crypt. Specimens were viewed under a TissueFAXS automatic scanning system, captured by a digital camera, and analyzed by the HistoQuest software (TissueGnostics, Vienna, Austria). Immunostaining analyses for CD44 and Ki67 were processed, and the antibodies used were as follows: anti-Ki67 (ab16667; Abcam, 1 : 200 dilution) and anti-CD44 (ab157107; Abcam, 1 : 2000 dilution) [15].

### 2.7. Safety of Probiotics

Blood samples were collected and cultured for possible bacteria. Specimens from the liver, spleen, and mesenteric lymph node were homogenized and seeded on MRS, BHI, and BIM-25 agar plates for bacterial investigation. Cultured bacteria from plate colonies were identified by the genomic sequence.

### 2.8. Statistical Analysis

Parametric data were presented as mean with standard deviation. Statistical significance was analyzed by one-way ANOVA. Data were analyzed with the IBM SPSS software (version 21.0; SPSS Institute, Chicago, USA). Values of *p* ≤ 0.05 were considered statistically significant.

## 3. Results

### 3.1. Effects of Lcr35 on Body Weight Changes and Diarrhea Scores of Mice with Intestinal Mucositis Induced by 5-FU

**Figure 1:**
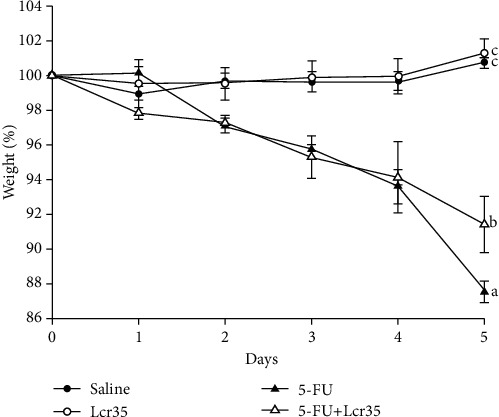
Daily body weight change in saline- or 5-FU-injected mice with/without probiotic Lcr35 administration. The mice were weighed daily, and the results of all groups were compared with those of 5-FU-saline groups for 5 days. In the control groups, the mice were injected with saline and administrated with saline or Lcr35. In the experimental groups, the mice were injected with 5-FU and administrated with or without Lcr35. Data on starting body weight are expressed as 100% from day 0. The body weight percentage sharply decreased from the 2^nd^ day after 5-FU treatment. The weight percentage of the 5-FU+Lcr35 group (91.41 ± 1.57%) was significantly decreased when compared to that of the 5-FU group (87.53 ± 0.63%) (*p* = 0.009). Statistical analysis was performed by one-way ANOVA.

All mice tolerated the experiments well, and no animal exhibited signs of adverse effects. No cachexia or mortality was found. The mice were weighed and compared daily. The average body weight increased in both the saline and Lcr35 groups (100.76 ± 0.27% and 101.31 ± 0.76%, respectively), though there was no significant difference between the 2 groups (Figure 1). In contrast, body weight in the 5-FU group decreased considerably. Body weight was sharply decreased from the 2^nd^ day in mice exposed to 5-FU when compared to the saline groups. Furthermore, in 5-FU-injected mice, the decrease in BW was significantly less severe following Lcr35 administrations when compared to those without probiotic administration (91.41 ± 1.57% vs. 87.53 ± 0.63%, *p* = 0.009).

**Figure 2:**
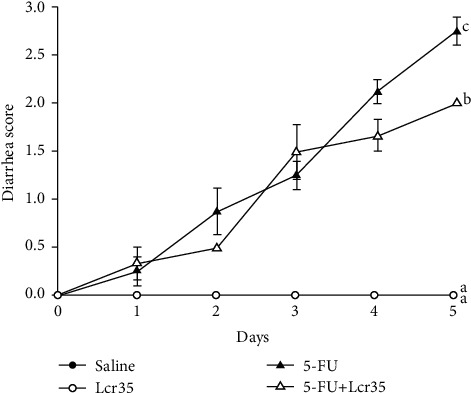
Diarrhea score change in saline- or 5-FU-injected mice with/without probiotic Lcr35 administration. The mice were recorded daily, and the results of all groups were compared with those in the 5-FU+saline group for 5 days. In the control groups, the mice were injected with saline and administrated with saline or Lcr35. In the experimental groups, the mice were injected with 5-FU and administrated with or without Lcr35. Diarrhea scores increased from the 1^st^ day after 5-FU treatment. Diarrhea scores of the 5-FU+Lcr35 group (2.00 ± 0.00) significantly decreased when compared to those of the 5-FU group (2.75 ± 0.14) (*p* = 0.001). The severity of diarrhea was attenuated after probiotic administration in the 5-FU+Lcr35 group. Statistical analysis was performed by one-way ANOVA.

Diarrhea scores of the mice were recorded and compared too. There was no diarrhea noted in both the saline and Lcr35 groups. On the contrary, remarkable diarrhea developed in the two 5-FU groups 24 hours later. However, diarrhea was relieved after Lcr35 administration (Figure 2). An improved diarrhea score in the 5-FU+Lcr35 group (2.00 ± 0.00) was found when compared to that in the 5-FU group (2.75 ± 0.14, *p* = 0.001) 5 days later.

### 3.2. Effect of Lcr35 on Proinflammatory Cytokine Production

**Figure 3:**
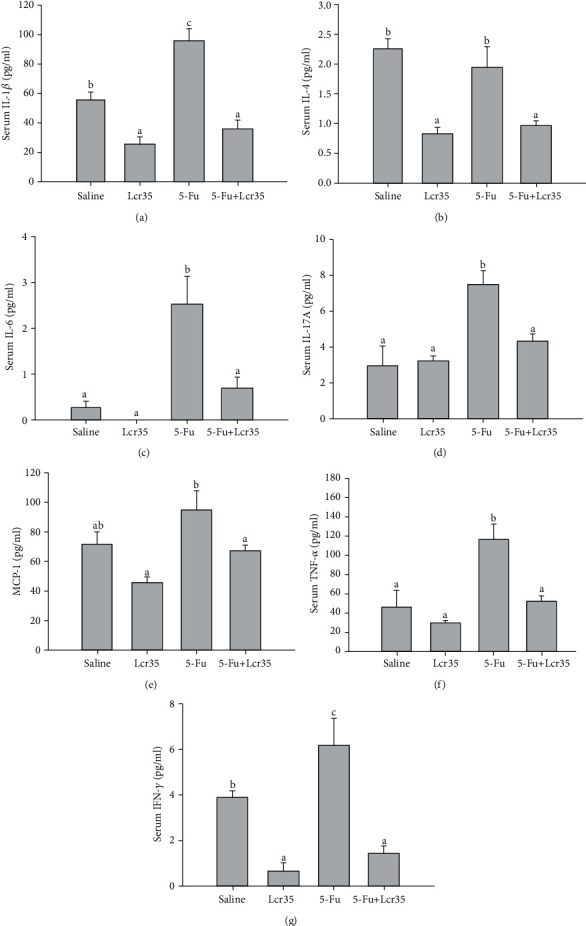
Upregulations of IL-1*β*, IL-4, IL-6, IL-17A, MCP-1, TNF-*α*, and IFN-*γ* in mucositis mice were shown after 5-FU injection. IL-6, IL-1*β*, and TNF-*α* serum levels were measured by the panel kit. (a) The serum IL-1*β* level in the 5-FU+Lcr35 group (35.8 ± 6.1 ng/ml) was significantly lower than that in the 5-FU group (95.8 ± 8.0 ng/ml) (*p* = 0.0001). (b) The serum IL-4 level in the 5-FU+Lcr35 group (0.97 ± 0.06 ng/ml) was significantly lower than that in the 5-FU group (1.95 ± 0.34 ng/ml) (*p* = 0.004). (c) The serum IL-6 level in the 5-FU+Lcr35 group (0.69 ± 0.23 ng/ml) was significantly lower than that in the 5-FU group (2.53 ± 0.62 ng/ml) (*p* = 0.003). (d) The serum IL-17A level in the 5-FU+Lcr35 group (4.35 ± 0.35 ng/ml) was significantly lower than that in the 5-FU group (7.50 ± 0.78 ng/ml) (*p* = 0.003). (e) The serum MCP-1 level in the 5-FU+Lcr35 group (67.1 ± 3.8 ng/ml) was significantly lower than that in the 5-FU group (94.1 ± 14.2 ng/ml) (*p* = 0.04). (f) The serum TNF-*α* level in the 5-FU+Lcr35 group (52.2 ± 4.9 ng/ml) was significantly lower than that in the 5-FU group (116.9 ± 14.7 ng/ml) (*p* = 0.001). (g) The serum IFN-*γ* level in the 5-FU+Lcr35 group (1.45 ± 0.31 ng/ml) was significantly lower than that in the 5-FU group (6.16 ± 1.21 ng/ml) (*p* = 0.0001). Statistical analyses were performed by one-way ANOVA.

The effect of *Lcr35* on proinflammatory cytokine production assays is shown in Figure 3. Serum levels of IL-1*β* (a), IL-4 (b), IL-6 (c), IL-17A (d), MCP-1 (e), TNF-*α* (f), and IFN-*γ* (g) were evaluated. Serum levels of these proinflammatory cytokines were significantly increased in the 5-FU group when compared to the saline group. This suggested a severe pattern of intestinal mucositis in mice. On the contrary, a significant reduction of expression levels of various cytokines was observed after administration of the probiotic: IL-1*β* (*p* = 0.001), IL-4 (*p* = 0.004), IL-6 (*p* = 0.003), IL-17A (*p* = 0.003), MCP-1 (*p* = 0.004), TNF-*α* (*p* = 0.001), and IFN-*γ* (*p* = 0.0001) when compared to the 5-FU group (Figures 3(a)–3(g)).

### 3.3. Flow Cytometry of T-Cell Subtypes

**Figure 4:**
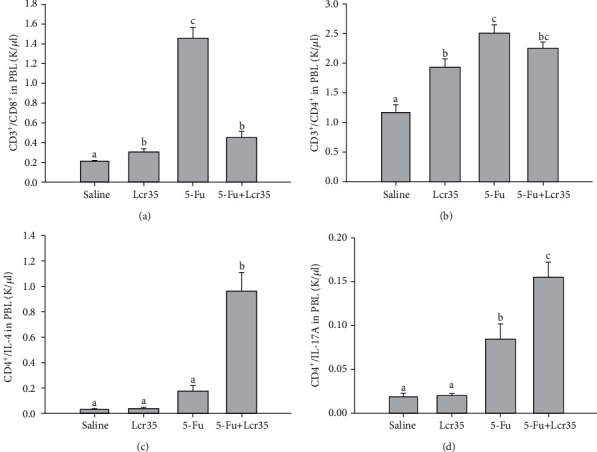
Effect of Lcr35 administration on the T lymphocyte count by flow cytometry analysis. (a) A tremendous rise of the CD3^+^/CD8^+^ lymphocyte count in the 5-FU group (1.45 ± 0.10 K/*μ*l) was observed when compared to that in the saline group (0.21 ± 0.01 K/*μ*l). The CD8 T lymphocyte count in the 5-FU+Lcr35 group (0.46 ± 0.04 K/*μ*l) was significantly lower than that in the 5-FU group (*p* = 0.0001). (b) A significant increase of the CD3^+^/CD4^+^ T lymphocyte count was found in the 5-FU group (2.50 ± 0.15 K/*μ*l) when compared to the saline group (1.16 ± 0.14 K/*μ*l). The CD3^+^/CD4^+^ T lymphocyte count in the 5-FU+Lcr35 group (2.25 ± 0.08 K/*μ*l) was lower than that in the 5-FU group though no significant difference was found (*p* = 0.194). (c) The CD4^+^/IL-4 T lymphocyte count in the 5-FU+Lcr35 group (0.96 ± 0.15 K/*μ*l) was significantly higher than that in the 5-FU group (0.18 ± 0.04 K/*μ*l) (*p* = 0.001). (d) A tremendous rise of the CD4+/IL-17A lymphocyte count in the 5-FU group was observed when compared to that in the saline group. The CD4^+^/IL-17A T lymphocyte count in the 5-FU+Lcr35 group (0.16 ± 0.02 K/*μ*l) was significantly higher than that in the 5-FU group (0.08 ± 0.02 K/*μ*l) (*p* = 0.004). Statistical analysis was performed by one-way ANOVA.

The effect of Lcr35 administration on T-cell subtypes is assessed and shown in Figure 4. We found that there was a tremendous rise of the CD3^+^/CD8^+^ lymphocyte count in the 5-FU group (1.45 ± 0.10 K/*μ*l) when compared to the saline group (0.21 ± 0.01 K/*μ*l) (Figure 4(a)). The CD8 T lymphocyte count in the 5-FU+Lcr35 group (0.46 ± 0.04 K/*μ*l) was significantly lower than that in the 5-FU group (*p* = 0.0001) (Figure 4(a)). Besides, there was a significant increase of the CD3^+^/CD4^+^ T lymphocyte count in the 5-FU group when compared to the saline group (Figure 4(b)). The CD3^+^/CD4^+^ T lymphocyte count in the 5-FU+Lcr35 group (2.25 ± 0.08 K/*μ*l) was lower than that in the 5-FU group (2.50 ± 0.15 K/*μ*l) though no significant difference was found (*p* = 0.194). The CD4^+^/IL-4 T lymphocyte count in the 5-FU+Lcr35 group (0.96 ± 0.15 K/*μ*l) was significantly higher than that in the 5-FU group (0.18 ± 0.04 K/*μ*l, *p* = 0.001) (Figure 4(c)). Similarly, there was a tremendous rise of the CD4^+^/IL-17A lymphocyte count in the 5-FU group when compared to the saline group. The CD4^+^/IL-17A T lymphocyte count in the 5-FU+Lcr35 group (0.16 ± 0.02 K/*μ*l) was significantly higher than that in the 5-FU group (0.08 ± 0.02 K/*μ*l, *p* = 0.004) (Figure 4(d)).

### 3.4. Effects of Lcr35 on Histological Changes and Intestinal Stem Cells (CD44 Stem Cells and Ki67 Proliferation)

**Figure 5:**
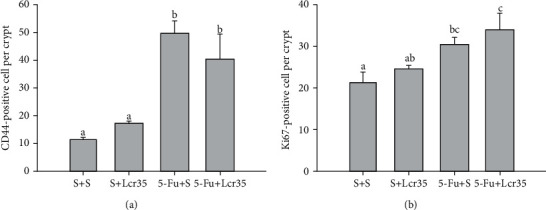
Effects of Lcr35 on CD44-positive stem cells and Ki67 proliferative cells in the intestinal mucosa. CD44-positive (a) and Ki67 (b) cells after staining were counted per crypt. 5-FU significantly stimulated the expression of CD44, and the expression was restored by administration of Lcr35, though not to the S+S or S+Lcr35 levels. 5-FU increased the number of Ki67-positive cells, but there were no significant differences between the 5-FU+S and S+S groups and the 5-FU+S and 5-FU+Lcr35 groups. Values were represented as mean ± SEM and were analyzed using one-way ANOVA.

**Figure 6:**
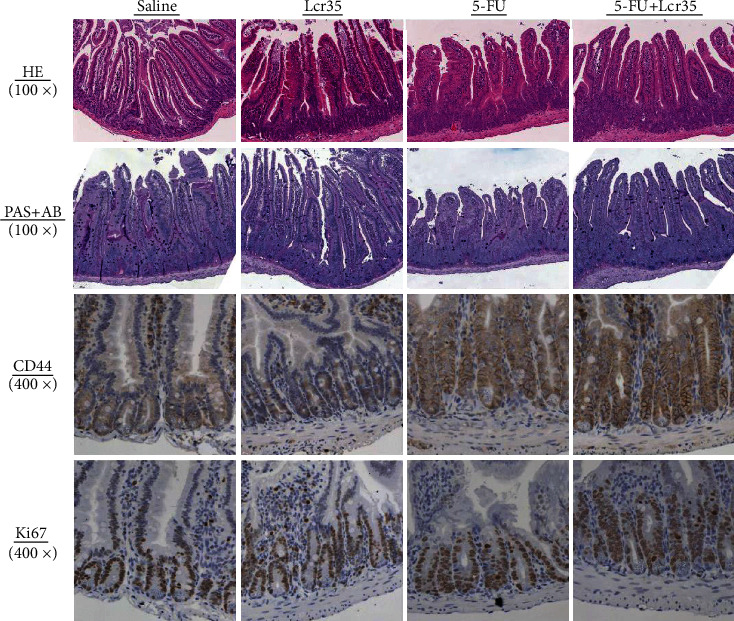
Representative histology of the jejunum showing villus height and crypt depth with hematoxylin and eosin stain. Microscopical findings of the intestinal mucosa from mice exposed to 5-FU-induced mucositis at the jejunum. Goblet cells were found in PAS+AB-stained sections. Intestinal stem cells CD44 markers and proliferation of crypt (Ki67 expression) were shown by IHC methods. CD44 analysis of intestinal stem cells and Ki67 immunolabeling of the jejunum from mice were assessed after 5-FU treatment. 5-FU significantly decreased villus height compared to the saline controls, and the effect was restored by probiotic treatment. 5-FU significantly lengthened the crypt depth of the intestine compared with the saline controls, and the effect was significantly restored by probiotic treatment.

Intestinal stem cells were represented by CD44 markers and Ki67 proliferative cells. CD44 markers and proliferation of crypts (Ki67 expression) were shown by IHC methods and are shown in Figures 5 and 6. CD44 analysis of intestinal stem cells and Ki67 immunolabeling of the jejunum from mice were assessed after 5-FU treatment. An increase in CD44 expression of intestinal stem cells and Ki67 proliferation were found in immunolabeled jejunal specimens from mice after the 5-FU challenge. We found that 5-FU significantly stimulated the expression of CD44. The expression was restored by administration of Lcr35, though not to the S+S and S+Lcr35 levels. 5-FU could increase the number of Ki67 proliferative cells, but there were no significant differences between the 5-FU+S and S+S groups and the 5-FU+S and 5-FU+Lcr35 groups.

Effects of Lcr35 on histological changes and stem cells in the intestinal mucosa from mice exposed to 5-FU are shown in Figure 6. 5-FU caused substantial changes in the intestinal mucosal layer, including the flattened epithelial layer, shortened villi, and lamina propria with inflammatory cell infiltration. The crypts looked small and narrow. No mitoses were found. Histochemistry stains for goblet cell amount, villus height, and crypt depth were assessed, and the results were similar to our previous study [10]. 5-FU significantly decreased villus height compared to the saline controls, and the effect was restored by probiotic treatment. Besides, 5-FU significantly lengthened the crypt depth of the intestine compared with the saline controls and the effect was significantly restored by probiotic treatment. The jejunum exhibited a significant decrease in total goblet cell numbers after treatment with 5-FU. This effect was alleviated by probiotic treatment too, resulting in a significant increase of goblet cell numbers compared with 5-FU controls (data not shown).

### 3.5. Safety and Translocation

Regarding the safety of probiotic administration, cultured bacteria were identified by the genomic sequence. We did identify 2 bacterial strains (*E. coli* str. *K*-*12*; *E. coli* O157:H7 str. *Sakai*; and *E. coli* UMN026) in the mesenteric lymph node in the saline group. Two bacterial strains (*Enterococcus dispar* ATCC 51266 genomic scaffold; *Enterococcus faecalis*; and *Enterococcus casseliflavus* EC20) were identified in the 5-FU group. However, no bacterial translocation was found in the samples of the blood, liver, and spleen tissues (Table 1).

## 4. Discussion

Intestinal mucositis is a frequently encountered adverse effect in cancer patients undergoing chemotherapy, and currently, there are no effective preventive and control measures [1, 4, 5]. 5-FU treatment was reported to affect the abundance of gut microbiota. In recent years, probiotics had been demonstrated with therapeutic effects in chemotherapy-induced mucositis. However, the results are inconsistent [14, 16]. We previously demonstrated that various *Lactobacillus* strains had shown beneficial effects on the mucosal barrier of intestines and could enhance tight junction integrity [9]. In this study, we hypothesize that the probiotics could preserve gut ecology, ameliorate inflammation, and protect epithelia by maintaining the tight junction integrity via immune modulations of enterocytes and intestinal stem cells. Our aim is to characterize these changes and to investigate the immune modulation effects and safety of probiotics via a 5-FU-induced intestinal mucositis mouse model.

In our mouse model study, body weight in the 5-FU group decreased considerably by day 2 after 5-FU administration. The body weight in the 5-FU+Lcr35 group decreased with less intensity when compared to that in the 5-FU group. On the contrary, we found that in those mice in the probiotic group, their degree in body weight loss was significantly lesser than those in the 5-FU and saline groups. Our results were similar to the findings of other studies in the literature [1, 17]. In our experiment, no diarrhea was noted in the saline and Lcr35 groups. However, marked diarrhea developed in the two 5-FU groups 24 hours later. We demonstrated that diarrhea scores improved significantly after oral Lcr35 administrations. Previous studies reported that more than one-third of the oncology patients undergoing chemotherapy experienced severe intestinal mucositis [18]. Benson et al. reviewed that the chemotherapeutic protocol containing 5-FU has been demonstrated with a higher risk for chemotherapy-induced diarrhea [19].

In our study, we showed that those mice in 5-FU+saline groups had significantly higher levels of proinflammatory cytokines. This suggested a severe pattern of intestinal mucositis in mice. However, the levels of these cytokines were significantly reversed after the administration of the probiotic in the 5-FU+Lcr35 group. We demonstrated that the protective effects of Lcr35 on 5-FU-induced mucositis were probably by triggering the Th1 immune response via downregulations of the cytokines IFN-*γ* and TNF-*α*. In an earlier study, Justino et al. reported that *Saccharomyces boulardii* lowered proinflammatory cytokine levels (TNF-*α*, IL-1*β*, and CXCL-1) in the rat jejunum and ileum induced by 5-FU [20]. The mechanism of *Saccharomyces boulardii*'s protective effect might be similar to the mechanism of Lcr35's action in our study. Up to date, the exact mechanism of chemotherapy-induced intestinal mucositis remains unclear. Previous studies had suggested that it involved a five-stage process [21–23]. Soares et al. suggested possible pathophysiology of mucositis development, including the generation of reactive oxygen species and the upregulation of proinflammatory cytokines causing further mucosal injury eliciting further tissue damage [24]. Few studies have assessed the effects of *Lactobacillus acidophilus* on inflammation. One of these studies found lower levels of leukocyte migration in animals treated with *Lactobacillus acidophilus* in a model of intestinal mucositis induced by irinotecan [25]. Several studies have reported reduced inflammatory effects using other probiotic species [20, 26].

We found that there was a tremendous rise of the CD3^+^/CD8^+^ lymphocyte count in the 5-FU group when compared to the saline groups. However, it was reversed after probiotic administration. The CD8 T lymphocyte count in the 5-FU+Lcr35 group was significantly lower than that in the 5-FU group. Besides, there was a significant increase of the CD3^+^/CD4^+^ lymphocyte count in the 5-FU group when compared to the saline groups. We suggested that the protective effect of Lcr35 on 5-FU-induced mucositis was by downregulations of the lymphocytes CD3^+^/CD8^+^ and CD8^+^/IFN-*γ*. The Lcr35 could also activate the T helper cells by stimulating the CD4^+^/IL-4^+^ cell maturation.

Similarly, there was a tremendous rise of the CD4^+^/IL-17A lymphocyte count in the 5-FU group when compared to the saline group. Interestingly, the level of the CD4^+^ T lymphocyte count further increased after probiotic administration. The amount of the CD4^+^/IL-17A lymphocyte count in the 5-FU+Lcr35 group was significantly higher than that in the 5-FU group. The Th17 immune response was demonstrated in CD4^+^/IL-17A^+^ lymphocyte activation in the 5-FU+Lcr35 group. Roles of CD4^+^/IL-17A lymphocytes on intestinal immunity and the pathophysiology of chemotherapy-induced mucositis have been investigated recently [27]. Edelblum et al. recently found that CD4^+^ T-cells, and in particular Th17 cells, were necessary to limit acute *Salmonella typhimurium* invasion in constitutively active myosin light chain kinase (CA-MLCK) mice. Studies in germ-free CA-MLCK mice showed that commensal bacteria are required for both CD4^+^ T-cell expansion and early protection against bacterial invasion [28]. The increase in CD4^+^/IL-17A^+^ lymphocyte activation in the 5-FU+Lcr35 group might suggest a further Th17 immune response.

To further explore the mechanism of probiotics, we also looked at the intestinal stem cells and crypt proliferation in this study. Intestinal stem cells represented by CD44 markers and crypt proliferation with Ki67 expressions were shown by IHC methods. Marked CD44 expression of intestinal stem cells and Ki67 proliferation were found in immunolabeled jejunal specimens after the 5-FU challenge. In our study, 5-FU significantly stimulated the expression of CD44 and the expression was restored by administration of Lcr35, though not to the S+S or S+Lcr35 levels. 5-FU could increase the number of Ki67^+^ cells, but there were no significant differences between the 5-FU+S and S+S groups and the 5-FU+S and 5-FU+Lcr35 groups. The actual role of probiotics on stem cell proliferation remains unclear and requires further investigation. Huang et al. showed that the Ki67 antigen plays a significant role in regulating cellular proliferation and has prognostic value in many types of cancer [29]. They demonstrated that high Ki67 expression was a significant prognostic factor for relapse, remote metastasis, and overall survival of patients. According to the studies, CD44 is one of the most common markers of cancer stem cells (CSCs) in gastric cancer, which has the potential for regeneration, initiation of tumor progression, and possibly development of metastasis [30]. Liang et al. demonstrated the prognostic significance of Ki67 and CD44s expression patterns in gastrointestinal stromal tumors [30]. Our results showed that 5-FU could stimulate stem cells and proliferation cells in a mouse model. It seems that stem cells and proliferation cells play roles such as immunomodulation effects in the pathophysiology of chemotherapy-induced intestinal mucositis. Actual mechanisms require further investigation. Ki67 and CD44s expressions might be considered markers in future studies on intestinal mucositis.

Athiyyah et al. investigated the probiotic effect of *Lactobacillus plantarum* IS-10506 in activating and regenerating leucine-rich repeat-containing G-protein-coupled receptor (Lgr) 5- and B lymphoma Moloney murine leukemia virus insertion region (Bmi) 1-expressing intestinal stem cells in rodents following *Escherichia coli* serotype O55:B5 lipopolysaccharide exposure [31]. Their results demonstrated that the probiotic *Lactobacillus plantarum* IS-10506 activated intestinal stem cells to counter inflammation and might be useful for maintaining intestinal health, especially when used as a prophylactic agent.

Probiotics are defined as living bacteria that can confer health benefits to the host. However, potential side effects, including sepsis development, presence of virulence factors, and translocation of live bacteria into local tissues, are possible [32, 33]. In the present study, we did identify 2 bacterial strains (*E. coli* str. K-12; *E. coli* O157:H7 str. *Sakai*; and *E. coli* UMN026) in the mesenteric lymph node in the saline group. Two bacterial strains (*Enterococcus dispar* ATCC 51266 genomic scaffold; *Enterococcus faecalis*; and *Enterococcus casseliflavus* EC20) were identified in the 5-FU group. However, no bacterial translocation was found in the samples of the blood, liver, and spleen tissues (Table 1). The risk of systemic infection with Lcr35 administration in this mouse model was not likely.

The pathophysiology of chemotherapy-induced mucositis is complex and most likely involves multiple different processes [34, 35]. In 2004, Sonis published the famous five-phase model theory to explain the pathophysiology of mucositis [21]. Different cytokines are responsible for the various stages. Proinflammatory cytokines such as IL-1*β* and TNF-*α* were also shown to play a role in amplifying the severity of chemotherapy-induced intestinal mucositis [36]. Over the past decade, this model has been built upon, with advances in our understanding in regard to the cell kinetics, epithelial junctions, inflammation, microbiome, and innate immune system [37].

Studies have shown that chemotherapy increases intestinal permeability, induces the generation of reactive oxygen species and proinflammatory cytokines, and modulates gut microbiota [21, 35]. In our previous study, we demonstrated that serum cytokine levels increased after 5-FU injection and were variously affected by probiotic administration in SCID/NOD mice, suggesting that innate immunity plays a role in the pathogenesis of intestinal mucositis [38]. IFN-*γ*, TNF-*α*, IL-1*β*, and IL-6 can promote inflammation when tissue injury or infection occurs. IL-1*β* plays a crucial role in the activation of the NF-*κ*B pathway, even working with TNF for a synergistic effect in kickstarting the inflammatory response of endothelial adhesion molecules. IL-4 acts on a variety of tissues, with receptors found in endothelial, epithelial, and even brain and liver cells in vitro [39], and it is originally secreted from Th2-type helper T-cells, mast cells, and basophils [40, 41].

In this study, we showed that Lcr35 could reduce levels of proinflammatory cytokines in the intestine in 5-FU-treated mice. Proinflammatory cytokines such as TNF-*α* and IL-6 contributed to the severity and maintenance of injury in intestinal mucositis [42], and IL-4 was found to participate as a proinflammatory cytokine in a model of 5-FU-induced intestinal damage [43]. Thus, the reduction of these cytokines suggested that the probiotic had strong anti-inflammatory activity.

We previously demonstrated that *Lactobacillus* was associated with the maintenance of the tight junction integrity [9]. However, the beneficial effects of probiotics on chemotherapy-induced mucositis were not consistent in the literature [36, 44]. In the current study, we determined the effect of probiotic treatment on the expressions of proinflammatory cytokines. We further explored the effects of probiotics on stem cells, T-cells, and cell proliferation. Our results showed convincing protective effect and safety of probiotics on the chemotherapy-induced mucositis. Recently, we successfully demonstrated that probiotics did modulate the abundance and diversity of gut microbiota of mice undergoing chemotherapy [13]. Previous studies in the literature seldom determined the effect of probiotic treatment on the expressions of proinflammatory cytokines. Furthermore, the safety of probiotic administration was rarely investigated.

There are several limitations to this study. One limitation is the small sample size of the mouse model used in this experiment, and we only investigated one probiotic strain. Besides, the mice used in this study were indeed normal mice without malignancy; we confessed the model could not mimic or represent the actual situation that happened in the clinical patients receiving chemotherapy. The duration of the experiment should be extended in future studies to evaluate the long-term influence of probiotics on microbiota modifications, rather than only the acute changes. Nevertheless, the greatest challenge for the animal model is the difficulty in translating results obtained from the current model to the wide range of human patient groups, with varying ages, cancer diagnoses, and treatments covering a wide range of drugs and doses of chemotherapy.

## 5. Conclusions

Our results showed that 5-FU causes intestinal inflammation mainly via Th1 and Th17 responses. 5-FU could stimulate stem cells and proliferation cells in a mouse model. Oral administration of probiotic Lcr35 can ameliorate chemotherapy-induced intestinal mucositis. This is the first study to analyze the immune modulation effects and safety of *Lactobacillus casei* variety *rhamnosus* on enterocytes and intestinal stem cells in a 5-FU-induced mucositis mouse model. The model therefore seems well suited to study the effects of different probiotics on chemotherapy-induced mucositis prior to performing human clinical studies.

## Figures and Tables

**Table 1:** Translocation of probiotics to the mesenteric lymph node, spleen, liver, and blood in 5-FU-treated mice fed with or without Lcr35. Cultured bacteria were from plate colonies and identified by the genomic sequence. ^a^*E. coli* str. K-12; *E. coli* O157:H7 str. *Sakai*; and *E. coli* UMN026 were identified. ^b^*Enterococcus dispar* ATCC 51266 genomic scaffold; *Enterococcus faecalis*; and *Enterococcus casseliflavus* EC20 were identified.

	Mesenteric lymph node	Spleen	Liver	Blood
S+S	2/4^a^	0/4	0/4	0/4
S+Lcr35	0/5	0/5	0/5	0/5
5-FU+S	2/4^b^	0/4	0/4	0/4
5-FU+Lcr35	0/5	0/5	0/5	0/5

Plate colony culture (bacteria positive/mouse number).

## Data Availability

All data generated or analyzed during this study are included in this published article. Further information are available from the corresponding authors on reasonable request.

## Abbreviations

5-FU: 5-Fluorouracil
Lcr35: *Lactobacillus casei* variety *rhamnosus*
IP: Intraperitoneally.
